# Infection Dynamic of Symbiotic Bacteria in the Pea Aphid *Acyrthosiphon pisum* Gut and Host Immune Response at the Early Steps in the Infection Process

**DOI:** 10.1371/journal.pone.0122099

**Published:** 2015-03-26

**Authors:** François Renoz, Christine Noël, Abdelmounaim Errachid, Vincent Foray, Thierry Hance

**Affiliations:** 1 Earth and Life Institute, Biodiversity Research Centre, Université Catholique de Louvain, Louvain-la-Neuve, Belgium; 2 Institut des Sciences de la Vie, Université Catholique de Louvain, Louvain-la-Neuve, Belgium; CSIRO, AUSTRALIA

## Abstract

In addition to its obligatory symbiont *Buchnera aphidicola*, the pea aphid *Acyrthosiphon pisum* can harbor several facultative bacterial symbionts which can be mutualistic in the context of various ecological interactions. Belonging to a genus where many members have been described as pathogen in invertebrates, *Serratia symbiotica* is one of the most common facultative partners found in aphids. The recent discovery of strains able to grow outside their host allowed us to simulate environmental acquisition of symbiotic bacteria by aphids. Here, we performed an experiment to characterize the *A*. *pisum* response to the ingestion of the free-living *S*. *symbiotica* CWBI-2.3^T^ in comparison to the ingestion of the pathogenic *Serratia marcescens* Db11 at the early steps in the infection process. We found that, while *S*. *marcescens* Db11 killed the aphids within a few days, *S*. *symbiotica* CWBI-2.3^T^ did not affect host survival and colonized the whole digestive tract within a few days. Gene expression analysis of immune genes suggests that *S*. *symbiotica* CWBI-2.3^T^ did not trigger an immune reaction, while *S*. *marcescens* Db11 did, and supports the hypothesis of a fine-tuning of the host immune response set-up for fighting pathogens while maintaining mutualistic partners. Our results also suggest that the lysosomal system and the JNK pathway are possibly involved in the regulation of invasive bacteria in aphids and that the activation of the JNK pathway is IMD-independent in the pea aphid.

## Introduction

As all living organisms, insects have to face a wide range of parasites and pathogens present in their environment. Unlike vertebrates, which are provided with an innate immunity coupled to a specific adaptive immunity to combat infection, insects depend solely on innate defense reactions, including the use of physical barriers together with cellular and humoral responses. These immune responses have been mostly described in the insect model *Drosophila melanogaster* [1,2], but have also been found to be inducible in other holometabolous insects [3–7]. The cellular immune system is based on immune-competent cells called hemocytes, which participate in phagocytosis and encapsulation of foreign intruders, and lead to melanization and clotting [8,9]. The humoral response is based on conserved signaling pathways leading to the release of effectors with antimicrobial activity. Once pattern recognition receptors (PRRs) bind and recognize pathogen-associated molecular patterns (PAMPs), signaling pathways such as Toll, immune deficiency (IMD), Jun-N-terminal kinase (JNK) and Janus kinase/signal transducers and activators of transcription (JAK/STAT) pathways are activated, resulting in the expression of antimicrobial peptides (AMPs) [10]. In addition to these immune pathways, insects can count on other defense mechanisms such as heat shock proteins (HSPs) production [11,12], nitric oxides release [13], reactive oxygen species (ROS) production [14] and the lysosomal system activation [15].

Symbiosis phenomenon is rather common in insects where many species have been shown to harbor symbiotic bacteria within their gut, tissues and/or cells [16]. By providing essential nutrients, such as amino acids and vitamins, some symbionts are obligate partners for their host [17–20]. These ones are generally confined in specialized host cells called bacteriocytes, stably maintained through host generations by vertical transmission, and often exhibit host-symbiont co-speciation and reductive genome evolution [21]. Thanks to their well-studied associations with a wide range of bacterial symbionts [22], aphids represent a valuable model system to study molecular interactions and immune responses of a host with both beneficial and harmful microorganisms. These sap-feeding insects typically harbor the obligate symbiont *Buchnera aphidicola* for delivering essential amino acids [23]. But in addition to their obligate partner, aphids, such as other insect groups, can also harbor symbionts which are facultative for their host in the sense that they can be responsible for various ecologically important traits under specific ecological conditions such as resistance against parasites [24,25], host plant usage influence [26], heat stress tolerance [27,28] and body color modification [29]. These facultative partners are vertically transmitted, although they can experience occasional horizontal transfers [30,31], and can be acquired from environmental sources [32–35]. Facultative symbionts resemble invasive pathogens in that they may invade various tissues types (including reproductive organs, gut and hemolymph), need to pass over host defense mechanisms to persist and multiply, and use similar infection mechanisms [36,37].

Interestingly, the in-depth screening of the aphid genome reveals that the pea aphid model *Acyrthosiphon pisum* lacks central genes for immune response as established in *D*. *melanogaster* and other investigated insects [38,39]. *A*. *pisum* lacks insect’s typical antimicrobial peptides, essential genes in the IMD pathway (including peptidoglycan recognition proteins (PGRPs)), has relatively few hemocytes [40,41] and displays only low lysozyme-like activity and phenoloxidase activation reactions [42]. Despite evidences that the pea aphid presents a reduced immune capacity compared to other insects, it remains unclear how mutualistic bacteria as well as pathogenic bacteria are perceived at the initial steps of an infection.

*Serratia symbiotica* is one of the most frequent facultative symbionts found in aphids [22] and belongs to a genus where many members have been described as pathogens of various invertebrates [43,44]. In aphids, *S*. *symbiotica* is mainly known for providing defense against environmental heat stress [27] and increasing host resistance to parasitoid wasps [24]. In the Cedar aphid *Cinera cedri*, *S*. *symbiotica* supplies the metabolic abilities of *B*. *aphidicola* for tryptophan synthesis and has been described as an obligate partner [45,46]. Recently, culturing attempts have been successful for the *S*. *symbiotica* CWBI-2.3^T^ strain [47] and other strains of this symbiont (A. Grigorescu, personal communication), which is quite remarkable, since most facultative insect symbionts are not possible to cultivate. The discovery of *S*. *symbiotica* strains able to grow outside their host makes feasible experimental infections at the gut level in order to understand the infection dynamic of symbiotic bacteria inside their new host and how they are perceived by its immune system early after ingestion.

For an effective infection, facultative symbionts should not be perceived as enemies, which would thus result in a moderate immune response to promote the establishment of a stable association [48]. On the contrary, infection by a pathogen should result in a stronger immune response, limiting the damage caused to the host. The first aim of our study was to test this hypothesis by comparing the immune responses of pea aphids infected by *S*. *symbiotica* CWBI-2.3^T^ and individuals infected by the entomopathogenic bacterium *Serratia marcescens* Db11 early in infection process. The second aim of our study was to determine whether the free-living *S*. *symbiotica* CWBI-2.3^T^ has a pathogenic effect and to monitor symbiont infection dynamic in *A*. *pisum* at the initial steps of the infection using fluorescence and qPCR approaches. We focused our experiments on the gut level because the oral route is one of the most widespread entries of microorganisms in animals [49]. In this study, we were able to bring out that, unlike *S*. *marcescens* Db11 for which a moderate immune response has been detected, the free-living *S*. *symbiotica* CWBI-2.3^T^ does not trigger an immune reaction and is able to multiply within the aphid gut without causing apparent damages to its new host.

## Materials and Methods

### Aphids and bacteria

#### Insect rearing

The pea aphid *A*. *pisum* was used in this study. All the experiments were performed with the Tucson-uninfected subclone provided by Nancy Moran (University of Texas). The Tucson-uninfected sub-colony was established in 2005 from the Tucson pea aphid originally collected from *Vicia faba* in Tucson (Arizona, 1999) through curing of *S*. *symbiotica* with heat-shock [27]. Aphids were reared on seedlings of *Vicia faba* at 20°C with a photoperiod of 16L:8D to ensure parthenogenetic reproduction.

#### Bacterial strains

The free-living CWBI-2.3^T^ *S*. *symbiotica* strain was used in this study. This strain was isolated from a natural *Aphis fabae* collected in Belgium in 2009 [47]. Pure cultures were performed at 20°C, 150 rpm in 863 medium (1% glucose, 1% yeast extract and 1% casein peptone). For pathogen infection, the entomopathogenic *S*. *marcescens* strain Db11 was used in this study. Db11 is a spontaneous streptomycin-resistant mutant of Db10 originally isolated from a moribund fly [50]. *S*. *marcescens* Db11 was grown in Lysogeny broth (LB) with streptomycin (100 μg/ml) at 37°C, 150 rpm.

### PCR detection of symbionts

Previous to the infection experiment procedure, the integrity of the Tucson-uninfected pea aphids was checked by diagnostic PCRs. DNA from individual aphids was extracted by using the QIAamp tissue kit (Qiagen). The PCR primers used for *S*. *symbiotica* detection are 16SA1 (5’-AGAGTTTGATCMTGGCTCAG-3’) and PASScmp (5’-GCAATGTCTTATTAACACAT-3’) [51]. The PCR reactions were performed in a final volume of 15 μl containing 1 μl of the template DNA lysate, 0.5 μM of each primer, 200 μM dNTP’s, 1X buffer and 0.625 unit of Taq DNA polymerase (Roche). The PCR reaction conditions consisted of 40 cycles of 95°C for 1 min, 55°C for 1 min 30 sec and 72°C for 1 min 30 sec. The PCR products were stained with ethidium bromide and visualized on a 1% agarose gel.

### Oral infection procedure

For the infection experiment procedure, bacterial solutions were prepared as described in [52]. The protocol was adapted in order to obtain three groups of aphids depending on the infection status and allowing comparative studies: (1) aphids infected by *S*. *symbiotica* CWBI-2.3^T^ (symbiont infection), (2) aphids infected by *S*. *marcescens* Db11 (pathogen infection) and (3) uninfected aphids (control). Briefly, when reaching an optical density (OD) of 0.5 at 600 nm during the logarithmic growth phase, the bacteria were centrifuged. The resulting pellets were washed with sterile PBS (Sigma) and resuspended in PBS in order to reach an optical density (OD) of 1 at 600 nm (corresponding to 9.7 × 10^7^ CFUs for *S*. *symbiotica* and 3.2 × 10^9^ CFUs for *S*. *marcesecens*, data not shown). Sterile PBS was used for control treatments.

To obtain synchronized aphid individuals, reproductive mature females were left in the dark for 24 h within sterile feeding chambers containing a small dish of artificial diet (provided by Viridaxis SA, Belgium) sealed with stretched parafilm. The produced progeny remained on this diet for three days (third instar) preceding the infection experiments. Third-instar aphid nymphs were fed on an artificial diet containing the bacteria for 24 h. 100 μl of bacterial solution (only sterile PBS for the control treatment) was mixed with 20 ml aphid diet (corresponding to approximately 10.4 × 10^4^ CFUs per ml of diet for *S*. *symbiotica* and 7.6 × 10^6^ CFUs per ml of diet for *S*. *marcescens*) and one drop of food-grade blue dye was added per 5 mL of diet to detect whether aphids had ingested the diet. After feeding, aphids were transferred on seedling of *V*. *faba*, except those devoted to survival monitoring which were reared within new sterile feeding chambers to facilitate the counting procedure.

### Survival curves

For each treatment, three sterile feeding chambers containing 15 aphids were monitored. Aphids were reared at 20°C with a photoperiod of 16L:8D. Survivors were counted every day for 7 consecutive days. The aphids alive after 7 days were considered as censored data in the analysis [53].

### Visualizing *S*. *symbiotica* in the aphid gut: *in situ* hybridization

To visualize general infection of *S*. *symbiotica* CWBI-2.3^T^, whole-mount *in situ* hybridization was performed, as previously described in [54]. At different times after the 24 h infection procedure (0, 5 and 10 days post-ingestion), randomly sampled aphids were placed in acetone for preservation and the first step of fixation. Insects were then placed in 70% ethanol for 30 min to soften the tissues. The legs were dissected to facilitate future visualization. The samples were fixed in Carnoy solution at room temperature overnight. After a thorough washing with 100% ethanol, the aphid samples were bleached in alcoholic 6% H_2_O_2_ solution to quench autofluorescence and incubated at room temperature until they were decolorized. Again, the samples were thoroughly washed with 100% ethanol. The following oligonucleotide probes were used for in situ hybridization: Cy5-ApisP2a (5′-Cy5-CCTCTTTTGGGTAGATCC-3′) targeting 16S rRNA of *B*. *aphidicola* and Cy3-PASSisR (5′-Cy3-CCCGACTTTATCGCTGGC-3′) targeting 16S rRNA of *S*. *symbiotica*. The tissue samples were hydrated with PBSTx containing 0.3% Triton X-100, incubated with hybridization buffer (20 mM Tris-HCl [pH 8.0], 0.9 M NaCl, 0.01% SDS, 30% formamide) containing 100 nM each of the probes and 0.5 μM SYTOX Green (Invitrogen) overnight, washed thoroughly with PBSTx, mounted in SlowFade antifade solution (Invitrogen), and observed under a Zeiss LSM 710 confocal microscope. Negatives controls consisted of uninfected aphids and no-probe staining.

### Quantitative PCR for *S*. *symbiotica* density

DNA extraction from single insects was conducted using a Qiagen DNeasy kit. The copy number of the symbiont genes in the DNA samples was measured by quantitative PCR on an Applied Biosystems Step One Plus machine (Applied Biosystems). *S*. *symbiotica* density in aphids was estimated at different time points after infection procedure: immediately after the infection procedure (0 day), 2, 4, 6, 8 and 10 days after ingestion. Primers specifically amplifying the *dnaK* gene from *S*. *symbiotica* as previously described by [27] were used (primers ApRF1 (5’-TGGCGGGTGATGTGAAG-3’) and ApRR1 (5’-CGGGATAGTGGTGTTTTTGG-3’)). The relative abundance of *S*. *symbiotica* was determined after normalization to the aphid single-copy elongation factor 1-alpha (*ef1-α*) gene by using the following primers: ApEF1-α 107F (5’—CTGATTGTGCCGTGCTTATTG—3’) and ApEF1-α 246R (5’—TATGGTGGTTCAGTAGAGTCC—3’). Each gene was amplified for 10 randomly sampled aphids. The PCR reaction mixture included 9.5 μl AB Power SYBR PCR mix (Life Technologies), 2.5 μl ddH_2_O, 1.5 μl of each primer (10 nM), and 5 μl DNA. The cycling conditions were: 15 min activation at 95°C followed by 40 cycles at 95°C for 15 s, at 60°C for 1 min, and 95°C for 15 s. Standard curves of both genes were prepared with 10-fold series of dilutions of genomic DNA at concentration of 10^2^, 10^3^, 10^4^, 10^5^, 10^6^, 10^7^ and 10^8^ copies/μl.

### Reverse transcription quantitative PCR (RT-qPCR)

The total RNA from five samples for each condition (symbiont infection, pathogen infection and control) each comprising four whole aphids was extracted by using the Qiagen RNA tissue kit following the manufacturer’s instructions immediately after the infection procedure (0h post-ingestion) and 17 h post-ingestion. cDNA was synthesized using a Qiagen QuantiTect reverse transcription kit following manufacture’s guidelines. The six targeted genes were chosen to cover some of the main defense mechanisms likely to be used when the aphid gut has to face Gram-negative bacteria intruders. The primer sequences used in this study are described in [39] and [55] and summarized in Table 1. Expression studies were carried out on Applied Biosystems Step One Plus machine (Applied Biosystems). The comparative C_T_ (ΔΔ C_T_) method was used to calculate relative gene expression levels with the aphid single-copy elongation factor (*ef1-α*) gene of *A*. *pisum* as endogenous control gene [56]. Finally, the variation in gene expression was determined after normalization to control treatment average at each time point, yielding the relative quantity (RQ) value. Each PCR reaction was performed in a final volume of 20 μL containing 10 μL AB Power SYBR PCR master mix, 50 nM of each primer and 10 ng cDNA. The PCRs were performed under the following conditions: 15 min activation at 95°C followed by 40 cycles at 95°C for 15 s, 60°C for 1 min, and 95°C for 15 s. For each sample, three separate reactions were carried out for each primer pair.

**Table 1:** List of genes and primers used in the study for quantitative PCR analyzed by quantitative real-time RT-PCR analysis.

**Gene symbol**	**Gene name**	**Putative function**	**Primer name**	**Primer pair (5’ to 3’)**	**References**
*Iap2*	Inhibitor of apoptosis 2	Imd/jnk pathway	Iap2_10F	TCGATGAACACAAACGTCACAA	
			Iap2_10R	GTTCACCAGTTTCCTTATGATTTTCAA	[39]
*Jra1*	Jun-related antigen	Imd/jnk pathway	Jra_1F	AAATCAAACTCGAAAGGAAAAGACA	
			Jra_1R	TTCGGCGGCATTTGGA	[39]
*Thm3*	Thaumatin 3	Antimicrobial peptide	Tha3_1F	GGGCAGGCAGGATTTGG	
			Tha3_1R	TTGGATCTTGTTCCCGCAAT	[39]
*Lys1*	Lysozime, i-type	Response to bacteria	Lysoz1_1F	CGCACAGGACTGCAACCA	
			Lysoz1_1R	GGATGGCCGCGTAATCAG	[39]
*HSP60*	Heat shock protein 60	Response to stress	hsp60_1F	GATGCAATGAACGACGAATATGTTA	
			hsp60_1R	CTGACAACTTTGGTTGGATCGA	[39]
*CTSL*	Cathepsin L	Response to bacteria	CTSL_F	ATCAGTACATCGCCTTCTCTTC	
			CTSL_R	GTACCGCAGACAATAACGTAG	[55]
*ef1-a*	Elongation factor	House-keeping gene	ApEF1-alpha 107F	CTGATTGTGCCGTGCTTATTG	
			ApEF1-alpha 246R	TATGGTGGTTCAGTAGAGTCC	[36]

### Statistical analyses

Nested-mixed ANOVA were used to analyze the density of *S*. *symbiotica* CWBI-2.3^T^ and the gene expression. The treatment of infection and the time post-ingestion were fixed main effects and replicate was a random effect. Tukey’s tests were used for *post hoc* comparisons. To analyze the effect of infection on the longevity of aphids, we used parametric survival analyses assuming a non-constant hazard function following a Weibull distribution. This model allowed for the incorporation of censored data. The significance of explanatory variables was assessed using z statistics. All statistical analyses were conducted using R 3.0.1 (R Development Core Team. 2010).

## Results and Discussion

### *S*. *symbiotica* CWBI-2.3^T^ has no pathogenic effect early after infection

**Fig 1:**
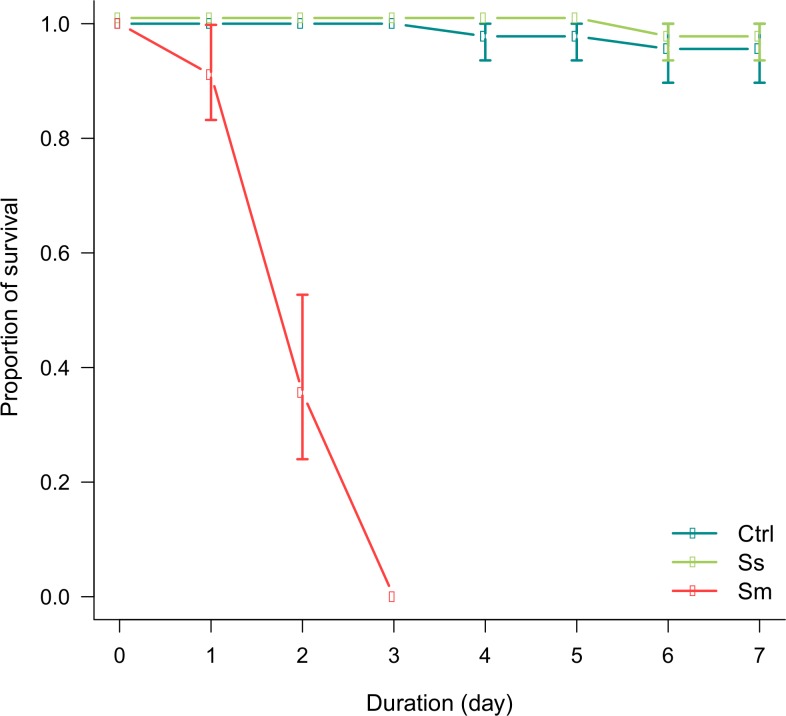
Survival of *A*. *pisum* aphids after bacterial ingestion. Graph show the survival of *A*. *pisum* aphids infected with *S*. *marcescens* Db11 (red), *S*. *symbiotica* CWBI-2.3^T^ (green) and uninfected aphids (blue). Error bars represent ± 95% CI.

Third instar aphids *A*. *pisum* were orally administrated either with PBS containing cultured *S*. *symbiotica* CWBI-2.3^T^ cells (symbiont infection) or with PBS containing cultured *S*. *marcescens* Db11 cells (pathogen infection) or with sterile PBS without bacteria (control). In our study, we chose to investigate the interaction between the pea aphid *A*. *pisum* and two closely related bacteria, *S*. *symbiotica* CWBI-2.3^T^ and *S*. *marcescens* Db11, as models to understand how aphids handle mutualistic bacteria in comparison to invasive pathogens at the early steps of an infection operated via oral route. When insects were administrated with *S*. *symbiotica* cells on diet, we observed no difference in term of mortality in comparison to the control (|z| = 0.581, *P* = 0.56). Almost all aphids infected with *S*. *symbiotica* CWBI-2.3^T^ were still alive and healthy at the end of one-week monitoring (Fig 1). On the other hand, the mortality of insects having ingested *S*. *marcescens* Db11 cells had increased significantly (|z| = 8.973, *P* < 0.001). They started to die within 24 hours after the ingestion and were all died within three days (Fig 1).

These observations suggest that *S*. *marcescens* Db11 is highly pathogenic for aphids, killing the insects rapidly after ingestion. Similar results have been observed in *D*. *melanogaster* where insects having ingested *S*. *marcescens* Db11 were killed within 6 days [44]. *S*. *marcescens* Db11 is highly virulent in insects and is able to rapidly pass the multiple physical and immune barriers protecting the digestive tract and penetrate the body cavity. Invasive capabilities and pathogenicity of *S*. *marcescens* Db11 involve proteases and chitinases targeting gut linings in invertebrates [44]. Our results for *S*. *symbiotica* CWBI-2.3^T^ infection suggest that, in the experimental conditions used in this study, the bacterium is not virulent, interacting in a nonpathogenic way when present in the gut of *A*. *pisum*. *S*. *symbiotica* strain CWBI-2.3^T^ was originally isolated from *A*. *fabae* [47] and is the first symbiotic bacterium of aphid with a free-living capacity whose genome has been sequenced [57]. While our observations support the idea that *S*. *symbiotica* CWBI-2.3^T^ is harmless when ingested by *A*. *pisum*, the nature of the interaction between this strain and aphids remains to be clarified. In addition to *S*. *symbiotica* CWBI-2.3^T^, several free-living *S*. *symbiotica* strains have been recently isolated (A. Grigorescu, personal communication), suggesting that these free-living forms of *S*. *symbiotica* could be frequent partners of aphids in addition to uncultivable endosymbiotic forms. Further nutritional and physiological studies are required in the future to identify biological roles of CWBI-2.3^T^ and other free-living *S*. *symbiotica* strains for aphid hosts, and to determine whether these bacteria play a mutualistic role, as the other *S*. *symbiotica* described until now [24,27,46], or whether these *S*. *symbiotica* strains with a free-living capacity should be considered as commensalistic partners. Analyses of the genome sequence of *S*. *symbiotica* strain CWBI-2.3^T^ should provide indications about the symbiotic nature of this strain.

### *S*. *symbiotica* CWBI-2.3^T^ multiply and persist in the aphid gut

**Fig 2:**
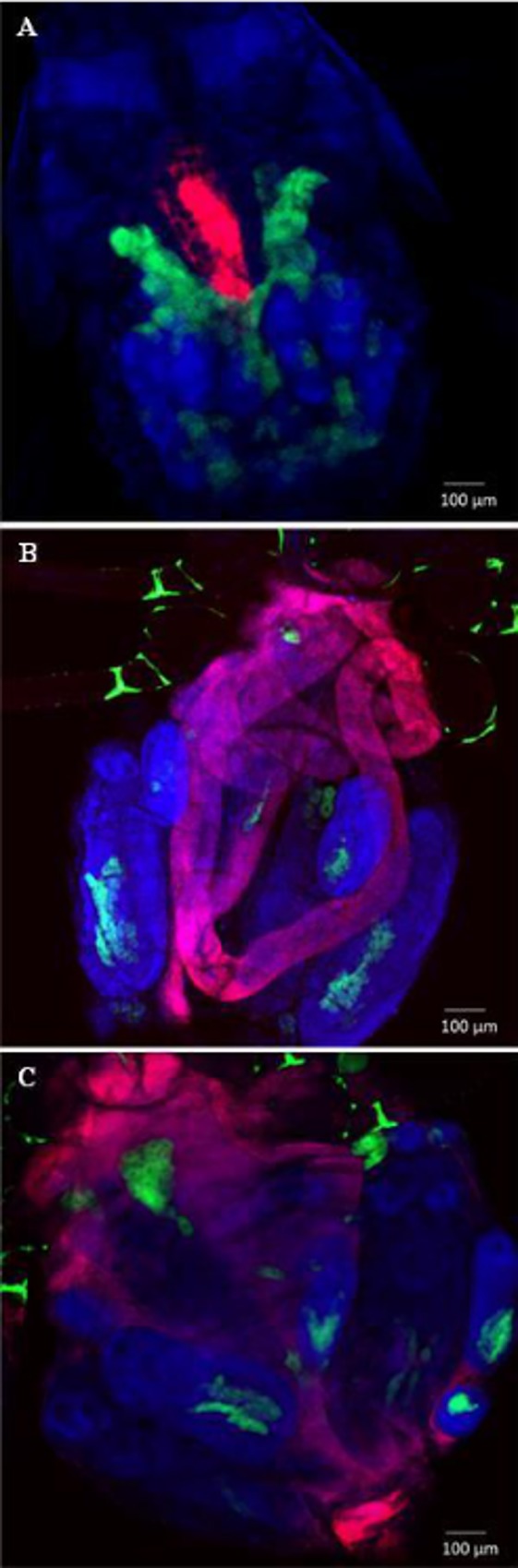
Whole mount fluorescence *in situ* hybridization of *S*. *symbiotica*-infected *A*. *pisum* 0 day post-ingestion (A), 5 days post-ingestion (B) and 10 days post-ingestion (C). Red Cy3 signals are *S*. *symbiotica* CWBI-2.3^T^, Green Cy5 signals are *B*. *aphidicola* and blue SYTOX Green signals are host tissues. (A) Red fluorescence in the midgut indicates ingestion of *S*. *symbiotica* CWBI-2.3^T^ after feeding of contaminated diet. (B and C) 5 days and 10 days post-challenge, red fluorescence is visible in the midgut as well as in the entire intestine.

When third instar aphids were administrated with *S*. *symbiotica* CWBI-2.3^T^ cells, infection of the digestive tract has been observed for a period of 10 days after inoculation. Using whole aphid fluorescence *in situ* hybridization (FISH), we determined that the free-living *S*. *symbiotica* CWBI-2.3^T^ strain was able to disseminate through the whole digestive tract of infected insects. In the early infection steps (0 day post-ingestion), the symbiont cells form an aggregate in the midgut (Fig 2A). 5 days after the infection, the bacteria can be found into the whole gut (Fig 2B). 10 days after ingestion, the bacteria are still present in large quantities and dispersed in the whole gut (Fig 2C).

**Fig 3:**
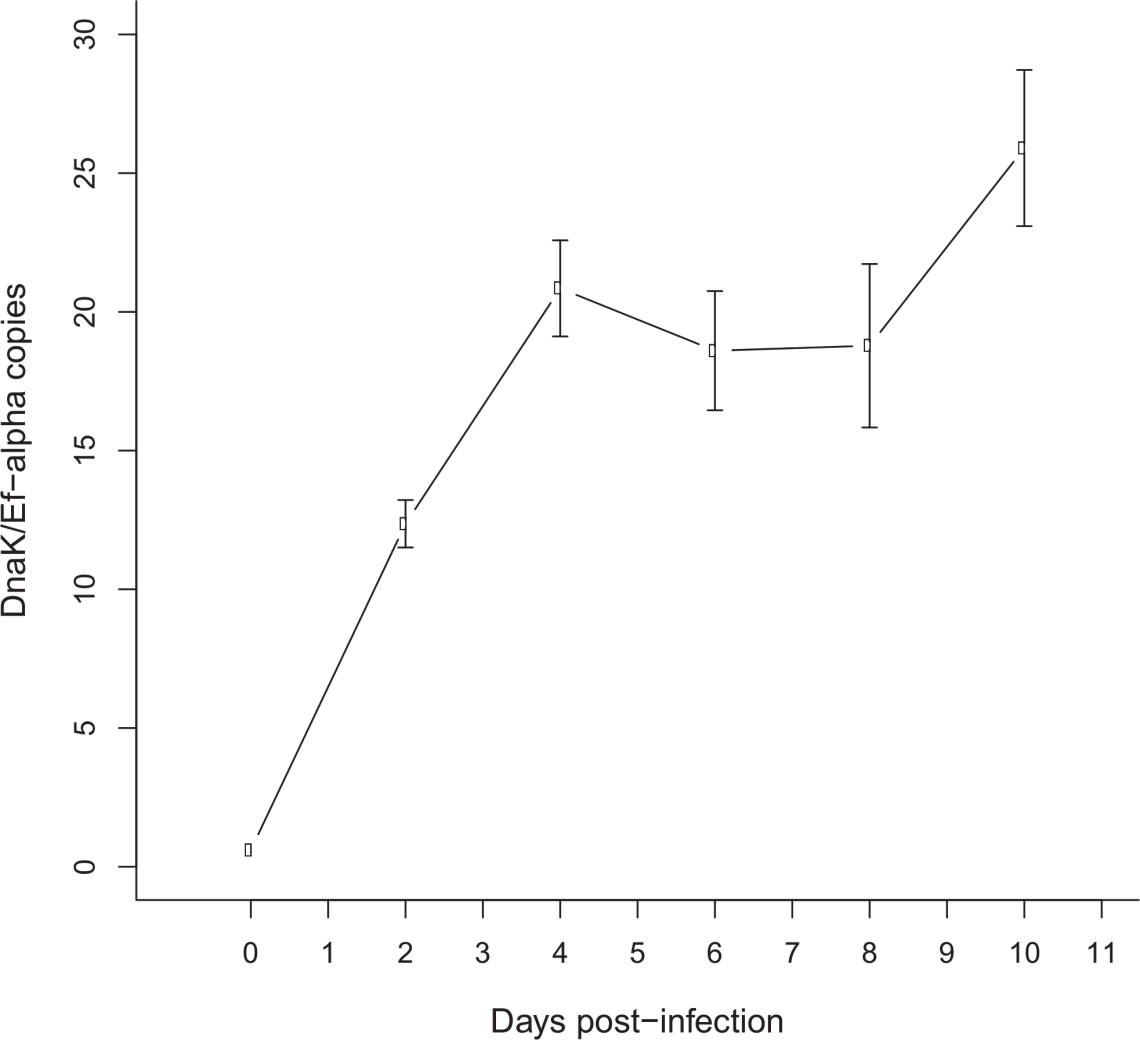
Infection dynamic of *S*. *symbiotica* CWBI-2.3^T^ during a period of ten days post-challenge. *S*. *symbiotica* CWBI-2.3^T^ densities are expressed in terms of *DnaK* gene copies per *ef1-α* gene copy. Points and bars indicate means and standard errors. Points sharing the same letters are not significantly different (p > 0.05) using post-hoc Tukey’s tests.

In addition to the visualization of gut infection using a fluorescence approach, we investigated the population dynamics of *S*. *symbiotica* CWBI-2.3^T^ in the gut during the 10 first days of the infection process with the quantitative PCR approach. We evaluated the *S*. *symbiotica* densities in terms of *dnaK* gene copies per *ef1-α* gene copy (Fig 3). This approach revealed that the symbiont titers in the infected insects exponentially increased as the host development proceeded, reaching a plateau 4 days after inoculation. From 8 to 10 days after ingestion, a slight increase of the symbiont titers is observed.

Our observations suggest that 1) the midgut provides an appropriate environment for *S*. *symbiotica* CWBI-2.3^T^ to multiply and accumulate before migrating and disseminating into the whole gut, 2) the free-living *S*. *symbiotica* CWBI-2.3^T^ strain is able to survive in the gut without apparent rejection from the host, 3) *S*. *symbiotica* CWBI-2.3^T^, which was originally hosted by *A*. *fabae*, it appears ubiquitous since it can found a refuge in the gut of an aphid species different from its original host. Population dynamics monitoring by the quantitative PCR approach supports FISH observations and reveals that the host colonization by the symbionts takes place rapidly during the first 4 days after ingestion. After 10 days, bacteria are still alive and are distributed through the whole intestine. *S*. *symbiotica* CWBI-2.3^T^ seems to be restricted to the aphid gut, suggesting the inability of the bacteria to go through the aphid gut epithelium to join the hemolymph. Bacteria, even pathogenic, are not always equipped to pass through the intestine. When *E*. *coli* K-12 infect aphids, the bacteria multiply in the whole intestine and kill the host within one week without going through the intestine epithelium [52]. Ongoing analyses of the CWBI-2.3^T^ genome should determine whether the bacteria possess the tools for intracellular invasion. It is also important to emphasize that virulence of bacteria can vary according to iron concentration, pH, temperature and other environmental factors [58,59]. Specific environmental conditions could ease the passage of the symbionts from the gut to the hemolymph and it cannot be excluded that environmental stresses weighing on aphids in natural conditions can promote such passages.

### *S*. *symbiotica* CWBI-2.3^T^ does not trigger an immune response while *S*. *marcescens* Db11 does

**Fig 4:**
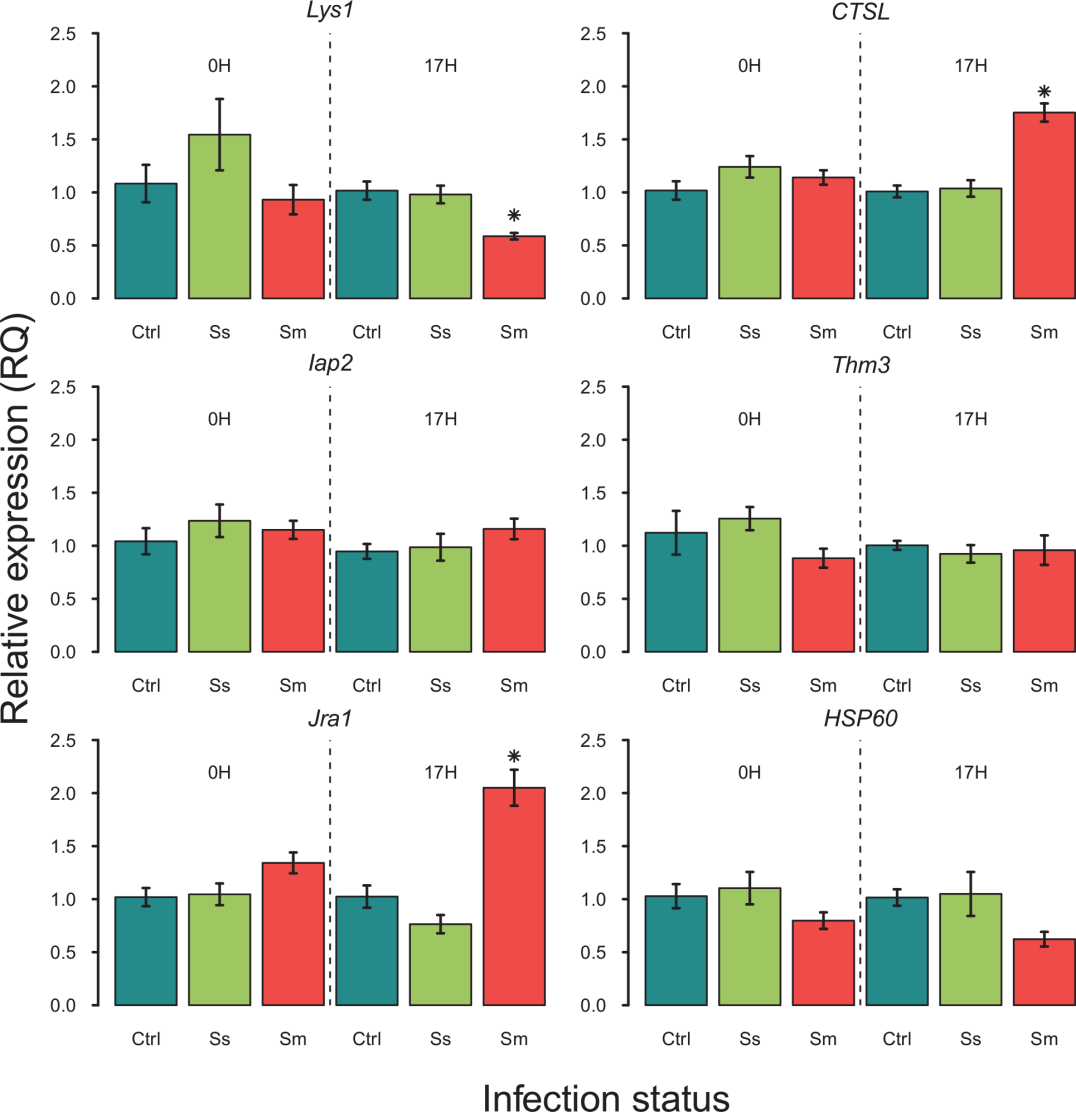
Quantitative real-time RT-PCR analysis of transcriptional levels of six selected *A*. *pisum* genes in response to bacterial infection. The mRNA levels of selected genes in infection with *S*. *symbiotica* CWBI-2.1^T^ (green), *S*. *marcescens* Db11 (red) were determined and are shown relative to their expression levels in untreated insects (blue). The expression level was normalized by comparative *C*_*T*_ (ΔΔ*C*_*T*_) method using *ef1-α* as reference gene. Columns and bars indicate means and standard errors.

In our study, we used real-time quantitative PCR to conduct an investigation of the expression of six recognition, signaling and response genes in aphids potentially expressed at the gut level after a bacterial challenging: *Iap2*, *Jra1*, *Thm3*, *Lys1*, *HSP60* and *CTSL*. Gene expression examining was achieved for three infection conditions (control, symbiont infection, pathogen infection) at two different times: immediately after ingestion (0 h post-ingestion) and 17 h post-ingestion (Fig 4).

We did not detect significant differences in normalized gene expression changes in comparison to the untreated group (control) for the six targeted genes 0 h post-ingestion for *S*. *symbiotica* CWBI-2.3^T^ and *S*. *marcescens* Db11 infections (*Iap2*: F = 0.48; *P* = 0.66; *Jra1*: F = 1.2, *P* = 0.44; *Thm3*: F = 0.51, *P* = 0.64; *Lys1*: F = 0.68, *P* = 0.57; *HSP60*: F = 0.74, *P* = 0.55 and *CTSL*: F = 0.83, *P* = 0.52). Neither did we find any effect of the infection treatment on the expression of *Iap2*, *Thm3* and *HSP60* 17 h post-ingestion (*Iap2*: F = 4.64, *P* = 0.12; *Thm3*: F = 0.09, *P* = 0.92; *HSP60*: F = 8.19, *P* = 0.061). These results are in accordance with other experimental studies and genomic data suggesting that the pea aphid displays no immune response or a weak immune response by contrast with other insect species when infected by microbial intruders [39,42,60]. Nevertheless, three genes of the panel of selected genes are differentially express relatively to the control 17 h post-ingestion for *S*. *marcescens* Db11: *Lys1* (post-hoc Tukey tests: *P* = 0.028), *Jra1* (*P* = 0.012) and *CTSL* (*P* = 0.007).

These differences in term of gene expression reveal that *S*. *marcescens* is able to trigger an immune response and that aphids can respond to the invasion of a microbial intruder. *Lys1* was chosen in our study as potential candidate gene for bacterial population regulation and host response because it can be expressed in insect gut as well as in hemolymph [10,61]. Moreover, *Lys1* is highly expressed in bacteriocyte harboring *Buchnera* [62] and [55] pointed out the potential role of i-type lysozymes in *Buchnera* density regulation. Lysozymes are bacteriolytic enzymes destroying bacterial cell walls by degrading peptidoglycan, thereby showing antibacterial activities and playing important defensive roles against bacterial infection [63]. In our study, *Lys1* appears to be slightly down-regulated in aphids infected by *S*. *marcescens* Db11 17 h post-ingestion (Fig 4). Conventionally, lysozymes have been regarded as inducible antimicrobial peptides highly expressed in response to bacterial infection [61]. However, down-regulation of *Lys1* has already been displayed in several bacteria-insects interactions. For example, expression of an i-type lysozyme gene is down-regulated in *Wolbachia*-infected pill bug *Armadillidium vulgare* [64] and the lysozyme gene *Rped-0033* of the bean bug *Riportus pedestris* is scarcely expressed in symbiotic insects in comparison to aposymbiotic insects [15]. To date, the role played by lysozymes in microbial infections and in the regulation of bacterial population in aphids remains unclear. In mammals, some bacterial pathogens have displayed an aptitude to down-regulate defense effectors to promote critical adherence and invasion into host epithelium [65]. It is therefore quite possible that *S*. *marcescens* Db11 moderately suppresses *Lys1* in aphids to promote host invasion. Nevertheless, as significant as they are, our results for *Lys1* have to be taken with caution due to the weak difference in term of gene expression. Studies targeting more lysozyme genes and conducted with other invasive bacteria are required to specify the role of these bactericidal proteins in aphids, both in mutualistic and pathogenic situations.

Cathepsin are lysosomal acidic proteases ubiquitously found in animals and function as digestive enzymes [66]. Although many cathepsin proteases are involved in intracellular protein turnover, cathepsin protease activities have been detected and characterized in the midgut of aphids and other insect species where they function as digestive enzymes [67]. Cathepsin-L proteases also participate in immunological processes and tissue remodeling during insect metamorphosis [68–70]. Nishikori et al. [55] pointed out the involvement of the lysosomal system in *Buchnera* degradation where cathespin L proteases could participate in the regulation of symbiont populations inside bacteriocytes. Over-expression of cathepsin L protease genes has also been detected in the midgut of the bean bug *R*. *pedestris* having ingested *Burkholderia* symbionts [15]. An up-regulation of cathepsin L and other proteins associated with protease activity have also been detected during tissue colonization at the initiation of the symbiosis between the bacterium *Vibrio fischeri* and its squid host [71]. These recent observations support the idea that cathepsins could play a crucial role in controlling symbiont populations. Both symbiotic and pathogenic bacteria have to avoid lysosomal degradation in order to establish an intracellular association. In our study, we observed a 2-fold up-regulation of *CTSL* in aphids infected by *S*. *marcescens* Db11 17 h post-ingestion (Fig 4). Over-expression of *cathepsin L* in aphids may function to digest bacteria and prevent pathogen invasion in cells and could therefore represent a mechanism by which the insect host would control invasive bacteria populations. Further studies are required to establish the biological role of cathepsins and other proteins associated with protease activity in the regulation of pathogen and symbiont populations in insects.

IMD and JNK signaling pathways are critical to fight Gram-negative bacteria in *Drosophila* and other insects [3,5,10]. Pea aphids appear to be missing many components of the IMD signaling pathway but have orthologs for most components of the JNK pathway, which play a role in antimicrobial peptide gene expression and cellular immune response [39]. Surprisingly, we observed no up or down-regulation of *Iap2*, but detected a 2-fold up-regulation of the *Jra1* gene for aphids infected by *S*. *marcescens* 17 h post-ingestion in comparison to the control (Fig 4). Although modest in comparison to what is observed in other insect species, this up-regulation of the *Jra1* gene suggests that 1) *S*. *marcescens* Db11 triggers some immune components of *A*. *pisum*, 2) the JNK pathways is active in pea aphids and undoubtedly plays a role in host defense against pathogenic bacteria, 3) in the pea aphid the activation of the JNK pathway is IMD-independent. The absence of response for *Iap2* comes to support this third hypothesis. Our study indicates for the first time that the JNK pathway could play a role in aphid-microorganisms interactions. Genes related to the JNK pathway could therefore be excellent candidates to explore the immune response of aphids facing bacterial intruders.

The absence of response for several genes and the weak response observed in our study for the targeting defense genes is not so surprising since the aphid immune system appear greatly limited in comparison to other well-studied insects, such as flies, mosquitoes, beetles and wasps. It is hypothesized that aphids compensate for a deficient immune system by symbiont-mediated host protection and a high reproduction rate [42,72]. It is also postulated that aphid-symbiont interactions may have led to the loss of immune pathways to accommodate long-term association with symbiotic bacteria and to facilitate the maintenance of symbionts [48]. The apparent reduced immunity in aphids could be the consequence of an evolutionary compromise preventing symbiotic partners’ elimination while fighting pathogens. A recent study on the pea aphid revealed that Macrophage Inhibitory Factors (ApMIFs), known as important regulators in innate immune response, are down-regulated in the presence or during the establishment of facultative symbionts but up-regulated upon challenge with pathogenic Gram-negative bacteria [48]. Moreover, this increase of expression levels of *ApMIFs* in the presence of pathogen is reduced in the presence of facultative symbionts, supporting the hypothesis of a fine-tuning of the immune response set-up for fighting pathogens while maintaining mutualistic partners. In accordance with this hypothesis, our observations show an absence of immune response to symbiotic infection for all the targeted genes, while some immune genes respond weakly but significantly when *A*. *pisum* has to cope with the pathogen *S*. *marcescens* Db11. This difference in terms of immune gene expression suggests that, unlike *S*. *marcescens*, *S*. *symbiotica* is not perceived as an intruder by the newly infected host. Our observations concerning the colonization of the aphid gut by *S*. *symbiotica* CWBI-2.3^T^ suggest that the symbiont is unable to go through the intestinal epithelium and most likely unable to enter the aphid cells, whereas *S*. *marcescens* Db11 can [44]. It cannot be excluded that the slight response perceived for *Lys1*, *Jra1* and *CTSL* 17 h after the ingestion of *S*. *marcescens* is the consequence of the pathogen entry into epithelial cells of the gut. Other investigations using other infection approaches, targeting more defense genes and involving other bacterial strains are necessary to determine which defense mechanisms are promoted by the infected aphid host to keep bacterial intruders, both mutualistic and pathogenic, under control.

## Conclusions

In conclusion, our study reveals that *S*. *symbiotica* CWBI-2.3^T^, originally isolated from the black bean *A*. *fabae*, interacts in a non-pathogenic way when ingested by a new host species, and colonizes the entire digestive tract within a few days. This demonstrates that the aphid gut constitutes a fitting environment for the development of this free-living *S*. *symbiotica* strain. These observations suggest the ubiquitous nature of *S*. *symbiotica* CWBI-2.3^T^, which seems able to find refuge in an aphid species different from the original host. The infection monitoring highlights that *S*. *symbiotica* CWBI-2.3^T^ seems not to be able to go through the gut epithelium and, unlike most of the *S*. *symbiotica* strains described as strictly intracellular partners, might have an extracellular life-style. The oral route is a preferred input for the bacteria, in particularly for symbiont acquired directly from the environment [32,73]. Nevertheless, we cannot exclude that *S*. *symbiotica* CWBI-2.3^T^ is also able to proliferate in other parts of the aphid body. Further experiments using microinjection technique to directly inject free-living *S*. *symbiotica* into the hemolymph would be appropriate to determine 1) whether *S*. *symbiotica* CWBI-2.3^T^ is able to survive in the hemolymph and avoid host immunity, 2) whether *S*. *symbiotica* CWBI-2.3^T^ can modulate cellar tropism of the infected host and be packed in bacteriocytes or sheath cells and, 3) whether *S*. *symbiotica* CWBI-2.3^T^ can be vertically transmitted from mother to offspring and establish a stable interaction in aphid populations.

According to the absence of variation in the expression level of the genes we have selected, the ingestion of *S*. *symbiotica* CWBI-2.3^T^ seems not triggering any aphid immune response. This finding suggests that *S*. *symbiotica* is not perceived as an antagonist by *A*. *pisum*, unlike *S*. *marcescens* Db11, for which a modest but significant response has been observed a few hours after ingestion. The absence of immune response for *S*. *symbiotica* could promote the development of a stable association between the symbiont and its new host. Regarding immune gene expression for *S*. *marcescens* Db11, we can hypothesize that invasive bacteria have to deal with the lysosomal system during aphid colonization and that components of the JNK pathway are functional defense mechanisms on which more attention should be paid in the future.

The discovery of free-living forms put forward the extraordinary diversity of *S*. *symbiotica* species, raising the question of the occurrence of these free-living strains in aphid populations and other insect species since *S*. *symbiotica* has been recently detected in ants [74–76]. To date, only the genus *Sodalis* presents an equivalent diversity with members described as obligate and facultative symbionts occurring both at intra- and extracellular levels [77] and pure-cultured systems have been already established for members of this genus, as well as for several members of the genus *Arsenophonus* [78]. Genomic and physiological analyses of *S*. *symbiotica* CWBI-2.3^T^ might provide insights into the mutualistic insect-bacterial symbioses and their evolutionary origin.
